# Optimisation of assessment of maximal rate of heart rate increase for tracking training-induced changes in endurance exercise performance

**DOI:** 10.1038/s41598-020-59369-6

**Published:** 2020-02-13

**Authors:** Maximillian J. Nelson, Clint R. Bellenger, Rebecca L. Thomson, Eileen Y. Robertson, Kade Davison, Daniela Schäfer Olstad, Jonathan D. Buckley

**Affiliations:** 1 Alliance for Research in Exercise, Nutrition and Activity (ARENA), University of South Australia, South Australia, Australia; 2 Adelaide Medical School and Robinson Research Institute, University of Adelaide, South Australia, Australia; 3 South Australian Sports Institute, Adelaide, Australia; 4 Polar Electro Oy, Kempele, Finland

**Keywords:** Interventional cardiology, Cardiovascular biology

## Abstract

The maximal rate of heart rate (HR) increase (rHRI), a marker of HR acceleration during transition from rest to submaximal exercise, correlates with exercise performance. In this cohort study, whether rHRI tracked performance better when evaluated over shorter time-periods which include a greater proportion of HR acceleration and less steady-state HR was evaluated. rHRI and five-km treadmill running time-trial performance (5TTT) were assessed in 15 runners following one week of light training (LT), two weeks of heavy training (HT) and 10-day taper (T). rHRI was the first derivative maximum of a sigmoidal curve fit to one, two, three and four minutes of R-R data during transition from rest to running at 8 km/h (rHRI_8 km/h_), 10.5 km/h_,_ 13 km/h and transition from 8 to 13 km/h (rHRI_8–13km/h_). 5TTT time increased from LT to HT (effect size [ES] 1.0, *p* < 0.001) then decreased from HT to T (ES −1.7, *p* < 0.001). 5TTT time was inversely related to rHRI_8 km/h_ assessed over two (B = *−5.54*, *p* = 0.04) three (B = *−5.34*, *p* = 0.04) and four (B = *−5.37*, *p* = 0.04) minutes, and rHRI_8–13km/h_ over one (B = *−11.62*, *p* = 0.006) and three (B = *−11.44*, *p* = 0.03) minutes. 5TTT correlated most consistently with rHRI_8 km/h_. rHRI_8 km/h_ assessed over two to four minutes may be suitable for evaluating athlete responses to training.

## Introduction

Athletes train to improve physical performance. This training can induce fatigue, but if combined with adequate recovery, it can promote adaptations that contribute to performance improvements^1^. This process of fatigue followed by positive performance improvement is known as functional overreaching^1^. However, if training is sustained for a prolonged period without adequate recovery it may result in performance decrements that can persist for days or weeks, with no subsequent improvement (i.e. non-functional overreaching) or the development of overtraining syndrome where performance can be impaired for weeks or months^2,3^.

Numerous studies have attempted to identify a physiological marker of fatigue to assist with informing adjustments to training to prevent development of non-functional overreaching or overtraining syndrome^3^, however to date no single marker capable of doing this has been identified^4^. High levels of post-exertional fatigue, and adaptations to physical training, can cause a shift in the function of the autonomic nervous system (ANS)^5^. As heart rate (HR) is regulated by the ANS, and is easily measured, it has been the focus of much research evaluating various HR parameters as markers of training adaptation. However, previously assessed HR parameters do not appear to be sufficiently sensitive to monitor both post-exertional fatigue-induced decreases in performance and training-induced improvements in performance^6,7^, possibly due to suboptimal and/or methodological inconsistencies in their assessment^6^.

Recently, it has been shown that the maximal rate of HR increase (rHRI), a marker of HR acceleration during the transition from rest to submaximal exercise, or during an increase in workload, correlates with fatigue-induced decreases in exercise performance, and training-induced increases in exercise performance, in both male^8–12^ and female athletes^13^. The mechanism by which rHRI correlates with exercise performance has not been determined, but Group III and IV skeletal muscle afferents project to the central nervous system and modulate cardiac function in relation to mechanical and metabolic status of the muscle^14,15^. Thus, it is possible that this afferent feedback might modulate rHRI in a manner that is consistent with changes in the mechanical and/or metabolic status of the muscle that affect exercise performance.

rHRI has previously been assessed by fitting a sigmoidal curve to five minutes of HR data collected during the transition from rest to exercise^9–12^, or during the transition from one workload to another^8^. Whilst this method has provided rHRI values that correlate with training-induced changes in exercise performance, it has not been established if the five minute duration for rHRI assessment is optimal. Assessing rHRI over a five minute period was initially selected^11^ to ensure that all individuals who underwent rHRI assessment would reach a steady-state HR during the assessment period. However, recent evidence^8^ suggests that reducing the duration of rHRI assessment may increase the sensitivity of rHRI for tracking changes in exercise performance. Bellenger, *et al*.^8^ monitored changes in exercise performance in competitive runners following strenuous training and recovery and found a moderate within-subject correlation between five-km running time trial performance and rHRI. Within that study, stronger correlations between rHRI and time trial performance were found in a subgroup of less fit athletes who demonstrated slower time trial performance. In those athletes, HR accelerated more slowly during the five minute period used for rHRI assessment, such that the acceleration component of the HR curve made up a larger proportion of the HR data used to calculate rHRI, with a lesser proportion of steady-state HR data contributing to the calculation. This suggests that the initial portion of the HR curve during rHRI assessment, when HR is accelerating, may be more important for tracking changes in exercise performance than the latter part of the curve when HR has typically reached steady state. Thus, the aim of this study was to determine whether analysis of rHRI over time periods shorter than five minutes improved the sensitivity of rHRI for tracking training-induced changes in endurance exercise performance.

## Methods

This study comprised a secondary analysis of data from a previous study that evaluated the ability of rHRI to track changes in exercise performance in 15 male runners/triathletes recruited in Adelaide, South Australia^8^. Testing was performed in the Sports Science laboratory of the South Australian Sports Institute between September 2013 and March 2014. Ethical approval was granted for the initial study by the University of South Australia Human Research Ethics Committee, and volunteers provided written informed consent prior to participating. The study protocol and findings are reported according to STROBE guidelines (https://www.strobe-statement.org).

The methods and eligibility criteria have been reported in detail elsewhere^8^, but briefly, participants had rHRI and five km treadmill time-trial (5TTT) performance assessed on three separate occasions: at the completion of a 7 day light training program (LT), which allowed a run-in period to enable participants to be well recovered from any prior exercise training; following a two week heavy training period (HT) which was intended to induce substantial fatigue; and following a 10 day taper period (T) to allow for recovery from HT and training adaptation to occur. To assess if there was a particular workload which would best track training-induced performance changes, rHRI was assessed during five minute running tests at three different exercise intensities: 8 km/h (rHRI_8 km/h_), which aimed to elicit ~65% of peak HR; 10.5 km/h (rHRI_10.5 km/h_), which aimed to elicit ~75% of peak HR; and 13 km/h (rHRI_13 km/h_), which aimed to elicit ~85% of peak HR. These running speeds were selected to elicit the desired HR values based on HR and running speed data from a previous study we performed in trained runners^16^. In addition, at the end of the 8 km/h test running speed was increased to 13 km/h, and continued for a further 5 minutes (rHRI_8–13 km/h_). The order of rHRI assessment was randomized at baseline and then held constant for each participant at subsequent visits. HR data were recorded during all tests using the RR-interval function of RS800CX HR monitors (Polar Electro Oy, Kempele, Finland).

rHRI was quantified by fitting a 5-parameter sigmoidal curve to data from the various rHRI assessment workloads. During the transition from rest to exercise the sigmoidal curve was fitted to R-R data recorded while participants were standing to the side of the treadmill during the 30 seconds prior to exercise onset and throughout the subsequent exercise. During the transition from running at 8 km/h to running at 13 km/h RR-data during the final 30 seconds of running at 8 km/h and throughout the subsequent exercise at 13 km/h were used for the sigmoidal curve fit. rHRI (bpm/sec) was determined as the first derivative maximum of this curve^8^.

To determine if reducing the duration of rHRI assessment would improve the sensitivity for tracking changes in exercise performance, rHRI was assessed for each workload (rHRI_8 km/h_, rHRI_10.5 km/h_, rHRI_13km/h_, rHRI_8–13 km/h_) over four, three, two and one minute time intervals, as opposed to the 5 minute intervals used for analysis of rHRI in previous studies^9–12^, including the previous analysis of the current data set in the primary study on which this secondary analysis is based^8^. The mean squared error of the residuals (MSE) and r^2^ were determined for the sigmoidal curves used to derive rHRI to identify if any durations of assessment resulted in better curve fits.

Random effect models were used for all statistical analyses using Stata/IC 15.0 (StataCorp, Texas, USA). Effect sizes were calculated for differences in 5TTT between LT, HT and T. Significance for all statistical tests was set at α = 0.05. All data are presented as mean ± standard deviation.

## Results

Participant characteristics and results of 5TTT are reported in detail elsewhere^8^. Briefly time taken to complete 5TTT increased from LT to HT (effect size [ES] 1.0, *p* < 0.001) and then decreased from HT to T (ES −1.7, *p* < 0.001), such that the time taken to complete 5TTT at T was significantly shorter (ES −1.3, *p* < 0.001) than at LT (LT 19:07 ± 2:11 min:sec, HT 19:28 ± 2:08 min:sec, T 18:43 ± 2:03 min:sec).

rHRI tended to not change significantly from LT to HT for most assessments (Tables 1 and 2), except for when it was assessed at 13 km/h, which resulted in significant increases when assessed over one, two and three minutes. Similarly, rHRI tended to not change from HT to T for most assessments, except at 8 km/h for periods of two, three and four minutes, and during the transition from 8 to 13 km/h when assessed over one minute (Table 1). rHRI increased from LT to T under most conditions (Table 1).

**Table 1:** rHRI determined from 4, 3, 2 and 1 minute periods of analysis at different running speeds.

rHRI analysis period	Running speed	LT rHRI (beats/min/sec)	HT rHRI (beats/min/sec)	T rHRI (beats/min/sec)	LT vs HT P value	LT vs T P value	HT vs T P value
4 min	10.5 km/h	3.83 ± 1.46	5.33 ± 3.04	6.45 ± 3.09	0.07	**0.002**	0.23
	13 km/h	3.85 ± 1.58	4.59 ± 1.80	4.89 ± 1.22	0.08	**0.02**	0.51
	8 km/h	4.99 ± 2.14	4.71 ± 2.12	6.23 ± 1.68	0.61	**0.04**	**0.009**
	8–13 km/h	1.45 ± 0.73	1.62 ± 1.10	2.14 ± 1.32	0.56	**0.03**	0.10
3 min	10.5 km/h	3.86 ± 1.48	5.29 ± 2.77	6.10 ± 2.63	0.05	**0.002**	0.33
	13 km/h	3.85 ± 1.42	4.73 ± 1.94	5.07 ± 1.26	**0.04**	**0.008**	0.48
	8 km/h	4.60 ± 1.85	4.71 ± 2.16	6.22 ± 1.72	0.88	**0.008**	**0.01**
	8–13 km/h	1.68 ± 0.86	1.37 ± 0.68	1.92 ± 1.02	0.32	0.44	0.07
2 min	10.5 km/h	3.73 ± 1.66	4.86 ± 2.34	5.58 ± 2.47	0.12	**0.01**	0.37
	13 km/h	3.77 ± 1.42	5.02 ± 1.91	5.23 ± 1.32	**0.005**	**0.002**	0.70
	8 km/h	4.20 ± 1.74	4.29 ± 2.04	5.84 ± 1.77	0.91	**0.005**	**0.006**
	8–13 km/h	1.54 ± 1.23	1.43 ± 0.84	1.97 ± 1.26	0.79	0.29	0.17
1 min	10.5 km/h	4.36 ± 2.10	5.96 ± 4.03	6.37 ± 3.16	0.15	0.07	0.78
	13 km/h	3.77 ± 2.17	5.33 ± 2.96	5.38 ± 2.37	**0.04**	**0.04**	0.98
	8 km/h	4.01 ± 2.08	4.44 ± 2.04	5.61 ± 2.64	0.59	**0.03**	0.09
	8–13 km/h	1.65 ± 1.41	1.19 ± 0.50	2.03 ± 0.89	0.19	0.29	**0.02**

Data presented as mean ± SD. Bold p-values indicate significant difference (p < 0.05) rHRI maximal rate of heart rate increase, *LT* post-light training, *HT* post-heavy training, *T* post-taper, *min* minute(s), *beats/min/sec* beats per minute per second, *km/h* kilometres per hour.

**Table 2:** Within subject relationships between five minute treadmill time-trial performance (5TTT) and rHRI determined from 4, 3, 2 and 1 minute periods of analysis.

Period of rHRI analysis	rHRI assessment	B-Coefficient	95% confidence interval for B-Coefficient	P value
4 min	rHRI_10.5 km/h_	−1.33	−5.31 to 2.65	0.51
	rHRI_13 km/h_	−2.34	−10.28 to 5.60	0.56
	rHRI_8 km/h_	−5.37	−10.60 to −0.14	**0.04**
	rHRI_8–13 km/h_	−9.48	−19.62 to 0.66	0.07
3 min	rHRI_10.5 km/h_	−1.11	−5.67 to 3.45	0.63
	rHRI_13 km/h_	−1.72	−9.15 to 5.70	0.65
	rHRI_8 km/h_	−5.34	−10.36 to −0.33	**0.04**
	rHRI_8–13 km/h_	−11.44	−21.55 to −1.33	**0.03**
2 min	rHRI_10.5 km/h_	−0.89	−5.63 to 3.86	0.71
	rHRI_13 km/h_	−0.14	−7.01 to 6.74	0.97
	rHRI_8 km/h_	−5.54	−10.68 to −0.40	**0.04**
	rHRI_8–13 km/h_	−4.78	−13.08 to 3.53	0.26
1 min	rHRI_10.5 km/h_	−0.09	−3.52 to 3.34	0.96
	rHRI_13 km/h_	−0.98	−5.37 to 3.41	0.66
	rHRI_8 km/h_	−4.53	−9.15 to 0.07	0.05
	rHRI_8–13 km/h_	−11.62	−19.86 to −3.38	**0.006**

*min* minute(s), *rHRI*_*10.5 km/h*_ maximal rate of heart rate increase in response to the 10.5 km/h treadmill running speed, *rHRI*_*13km/h*_ maximal rate of heart rate increase in response to the 13 km/h treadmill running speed, *rHRI*_*8km/h*_ maximal rate of heart rate increase in response to the 8 km/h treadmill running speed, *rHRI*_*10km/h*_ maximal rate of heart rate increase in response to the 10 km/h running speed, *rHRI*_*13km/h*_ maximal rate of heart rate increase in response to the 13 km/h running speed, *rHRI*_*8–13km/h*_ maximal rate of heart rate increase in response to the transition from 8 to 13 km/h.

rHRI_8 km/h_ was negatively related to 5TTT performance when assessed over durations of two, three and four minutes, with the strength of relationship being similar for each assessment duration, such that each one beat/min/sec increase in rHRI was associated with a 5.3–5.5 second reduction in time taken to perform the 5TTT (Table 2). Significant negative relationships were also found between 5TTT performance and rHRI_8–13 km/h_ when assessed over one and three minute periods, but not over two or four minute periods (Table 2). For rHRI_8–13 km/h_, each one beat/min/sec increase in rHRI was associated with an 11.4–11.6 second reduction in time taken to complete 5TTT. No other assessments of rHRI were significantly associated with 5TTT performance (Table 2).

Curve fit parameters for the sigmoidal curve fits used to determine rHRI are presented in Table 3. For the rHRI which correlated with 5TTT, rHRI_8 kmh_ demonstrated similar MSE across two, three and four minute assessments at each of LT, HT and T, but increasingly strong *r*^2^ as the duration of assessment decreased from four to two minutes. For rHRI_8–13 km/h_ the one minute duration of assessment demonstrated the lowest MSE and similar *r*^2^ across all time points (i.e. LT, HT and T).

**Table 3:** Curve fit parameters from sigmoidal curves used to determine rHRI over differing time intervals at different training stages.

		4 min		3 min		2 min		1 min	
		MSE	*r*^2^	MSE	*r*^2^	MSE	*r*^2^	MSE	*r*^2^
rHRI_8 km/h_	LT	10.96 ± 5.41^a,b^	0.922 ± 0.041^c^	9.71 ± 3.63^b^	0.940 ± 0.033^b^	10.46 ± 4.45^b^	0.952 ± 0.025^a^	12.85 ± 6.62^a^	0.953 ± 0.025^a^
	HT	9.99 ± 9.22^b^	0.937 ± 0.075^b^	11.06 ± 10.58^b^	0.944 ± 0.067^a,b^	11.35 ± 10.68^a,b^	0.956 ± 0.052^a^	13.44 ± 9.33^a^	0.957 ± 0.036^a^
	T	10.70 ± 4.84^b^	0.928 ± 0.041^b^	10.79 ± 5.20^b^	0.941 ± 0.032^b^	11.18 ± 5.74^b^	0.959 ± 0.018^a^	13.65 ± 8.73^a^	0.960 ± 0.018^a^
rHRI_8–13 km/h_	LT	3.68 ± 1.36^b^	0.968 ± 0.017^b^	3.34 ± 1.55^b^	0.973 ± 0.016^a,b^	3.06 ± 1.98^b^	0.977 ± 0.018^a,b^	3.30 ± 2.44^a^	0.970 ± 0.022^a^
	HT	3.64 ± 2.03^b^	0.959 ± 0.026^b^	2.60 ± 2.33^a^	0.976 ± 0.018^a^	2.56 ± 2.08^a^	0.979 ± 0.013^a^	1.99 ± 1.04^a^	0.980 ± 0.011^a^
	T	3.78 ± 2.09^c^	0.967 ± 0.022^c^	3.42 ± 2.08^b,c^	0.974 ± 0.016^b^	3.07 ± 1.71^a,b^	0.980 ± 0.010^a,b^	2.49 ± 1.10^a^	0.980 ± 0.009^a^
rHRI_10.5 km/h_	LT	10.14 ± 6.53^a,b^	0.946 ± 0.064^b^	8.36 ± 3.24^b^	0.968 ± 0.018^a^	9.14 ± 3.96^b^	0.972 ± 0.015^a^	13.03 ± 7.42^a^	0.960 ± 0.028^a,b^
	HT	8.26 ± 4.65^b^	0.965 ± 0.024^b^	9.68 ± 6.08^a,b^	0.966 ± 0.025^a,b^	9.89 ± 6.42^a,b^	0.974 ± 0.017^a^	11.44 ± 8.38^a^	0.973 ± 0.018^a^
	T	8.38 ± 5.07^b^	0.962 ± 0.029^b^	8.70 ± 5.93^b^	0.969 ± 0.026^b^	8.07 ± 5.69^b^	0.979 ± 0.013^a^	10.32 ± 8.57^a^	0.979 ± 0.012^a^
rHRI_13 km/h_	LT	7.02 ± 2.26^b^	0.980 ± 0.008^b^	7.60 ± 2.62^b^	0.982 ± 0.006^b^	9.67 ± 4.13^c^	0.981 ± 0.008^b^	14.62 ± 7.92^a^	0.973 ± 0.014^a^
	HT	7.35 ± 4.20^b^	0.978 ± 0.015^b^	7.49 ± 4.61^b^	0.982 ± 0.014^b^	9.16 ± 6.17^b^	0.982 ± 0.013^b^	14.53 ± 11.58^a^	0.974 ± 0.022^a^
	T	7.19 ± 2.79^b^	0.978 ± 0.013^b^	6.89 ± 2.42^b^	0.984 ± 0.006^a^	8.05 ± 3.54^b^	0.986 ± 0.006^a^	11.29 ± 6.64^a^	0.982 ± 0.010^a,b^

Values on the same row with different letters are significantly different (p < 0.05). *min* minute(s), *MSE* mean squared error of residuals, *LT* post-light training, *HT* post heavy training, T post taper, *rHRI*_*8 km/h*_ maximal rate of heart rate increase in response to the 8 km/h treadmill running speed, *rHRI*_*10 km/h*_ maximal rate of heart rate increase in response to the 10 km/h running speed, *rHRI*_*13 km/h*_ maximal rate of heart rate increase in response to the 13 km/h running speed, *rHRI*_*8–13 km/h*_ maximal rate of heart rate increase in response to the transition from 8 to 13 km/h.

## Discussion

This study used differing time periods of analysis of rHRI in an attempt to determine the time period that was most sensitive for tracking training-induced changes in exercise performance. The main finding was that assessment of rHRI during the transition from rest to running at 8 km/h (i.e. rHRI_8 km/h_) most consistently tracked 5TTT performance. Significant negative relationships were found between 5TTT performance and rHRI_8 km/h_ when rHRI_8 km/h_ was assessed over two, three and four minutes. The sigmoidal curves used to determine rHRI_8 km/h_ demonstrated similar MSE when assessed over each time period from two to four minutes, but the *r*^2^ values increased as the assessment duration decreased. This suggests that assessments over shorter time intervals provided better curve fits, but this did not appear to influence the strength of relationship between rHRI_8 km/h_ and 5TTT as the B values were similar (i.e. 5.3–5.5) regardless of whether rHRI_8 km/h_ was assessed over two, three or four minutes. rHRI assessed during the transition from light to higher intensity exercise (i.e. rHRI_8–13 km/h_) exhibited superior curve fit parameters compared with rHRI_8 km/h_ (lower MSE and higher *r*^2^), and was more strongly associated with 5TTT performance when assessed over one and three minute time periods, but was not significantly related to 5TTT when assessed over two or four minute time periods. It is not clear why rHRI_8–13 km/h_ was not related to 5TTT performance when assessed over two and four minute time periods, but the lack of consistency in relationship with 5TTT argues against the use of rHRI_8–13 km/h_ for tracking performance compared with rHRI_8 km/h_ which tracked 5TTT performance more consistently across a range of time periods. Furthermore, the second phase of rHRI_8–13 km/h_ involved running at relatively high intensity, and it would be preferable for any test evaluating how an athlete is responding to training require only low intensity exercise to avoid exacerbating any underlying fatigue^17^. Therefore, evaluation of rHRI_8 km/h_ would seem more appropriate.

The training protocol resulted in large changes in 5TTT performance across the different study periods. The heavy training loads performed during HT resulted in a considerable increase in time taken to run 5TTT between LT and HT (ES 1.0), indicating the presence of substantial fatigue. Run time for 5TTT then reduced substantially (ES −1.7) between HT and T, when the training load was tapered to facilitate adaptation to the prior training. Overall, from the start of the study (i.e. LT) to the end of the taper period there was a large reduction in the time taken to run 5TTT (ES −1.3). This indicates that rHRI was able to track reductions in exercise performance resulting from heavy training, and improvements in exercise performance due to adaptation to training.

The finding of the strongest relationships between rHRI_8 km/h_ and 5TTT performance when rHRI was assessed over shorter time periods (i.e. two to four minutes) is consistent with the results of Bellenger, *et al*.^8^. Bellenger, *et al*.^8^ found that when rHRI was determined from five minutes of HR data, there was a stronger relationship with 5TTT in a sub-group of less well-trained athletes whose HR took longer to reach steady-state. The longer time to reach a steady-state HR in those athletes meant that the acceleration component of the HR curve represented a greater proportion of the data used to determine rHRI. This is consistent with the present study where analysis of rHRI over shorter time periods meant that a greater proportion of the data represented the acceleration component of the HR curve. Taken together, these data suggest that the accelerating component of the HR curve contains the information which allows rHRI to track exercise performance, and therefore durations of rHRI assessment which contain a greater portion of HR acceleration may be more sensitive to changes in exercise performance.

The ability for rHRI to track both fatigue-induced performance decrements, and improvements in exercise performance when positive adaptations occur, contrasts with previous research that has investigated other HR markers – such as HR variability^5^ or HR recovery^18^. HR variability and HR recovery have been unable to demonstrate an ability to track both increases and decreases in performance resulting from changes in training load. Consequently a recent review^7^ suggested that HR variability and HR recovery are not sufficiently sensitive to be used to monitor performance-based responses to training. The findings of the present study suggest that rHRI may be useful in this regard.

It is unclear why rHRI_10.5 km/h and_ rHRI_13 km/h_ did not correlate with 5TTT performance. It did not appear to be due to differences in the ability to accurately fit curves to the HR data as the curve fit parameters were similar to those for the other methods of assessing rHRI that were evaluated. Instead, rather than differences in the curve fitting, there may be a physiological basis for the differential ability for rHRI to track exercise performance at different exercise intensities. HR changes during exercise are mediated, in part, by afferent feedback from mechano- and metabo-sensitive type III/IV afferent neurons in skeletal muscle which relay information to the cardiovascular centre in the brain stem on the metabolic and mechanical status of the muscle, resulting in modification of sympathetic and parasympathetic outputs to the heart to alter HR^15^. The transition from rest to running at 10.5 and 13 km/h speeds for determining rHRI_10.5 km/h_ and rHRI_13 km/h_ would likely have resulted in large and sudden acute changes in the metabolic environment within the muscle, which would have altered feedback from the mechano- and metabo-sensitive type III/IV afferent neurons^14,15^. These acute local effects may have dominated afferent feedback, thus altering rHRI in a way that reflected more the acute effect of the exercise rather than the chronic mechanical/metabolic status of the muscle. Transitioning from rest to running at 8 km/h on the other hand would be less likely to cause as large acute metabolic changes within the muscle, and afferent signals to the cardiovascular centre in the brain stem to alter HR would therefore more likely reflect the chronic mechanical/metabolic status of the muscle in terms of how it has responded to training. In this way, how the muscle is responding to training may influence both the rHRI response and exercise performance. Therefore, we are proposing the rHRI represents an indirect index of the physiological status of the muscle, and thus its readiness to perform exercise, which is communicated via type III/IV afferent neurons. Future research should aim to confirm the underlying mechanisms which cause rHRI to correlate with training-induced changes in exercise performance.

This study was limited by the fact that the data were only collected on male athletes, and it is unknown if shortening the duration of assessment would have the same effect in female athletes. However, given that a previous study by Nelson, *et al*.^13^ confirmed that rHRI correlates with training induced changes in exercise performance in female athletes, it is likely that female athletes may exhibit a similar response. Regardless, rHRI appears to correlate with endurance exercise performance when athletes are in different states of physiological readiness as a result of changes in training load which induce fatigue and reduce performance, or allow for recovery and adaptation with improved performance. Thus, rather than having to perform maximal exercise tests to assess changes in athlete performance, in order to determine how they are responding to changes in training load, rHRI could be assessed using submaximal running (i.e. 8 kmh) to provide insight into how an athlete is responding to training. The test could be performed as part of a warm-up prior to training, or as part of a cool-down following training, and the information obtained would be compared to a reference point when rHRI had been previously assessed (e.g. when an athlete was well recovered and performing well). The information obtained could be used to guide the training prescription in order to optimise performance at selected time points. For example, if rHRI were slowed, suggesting that an athlete has not recovered from prior training, training loads may be reduced to facilitate recovery. Alternatively, if rHRI were faster, suggesting that an athlete is responding well to training and performance will be improved, the training load may be increased to facilitate further adaptation.

In conclusion, assessment of rHRI, which provides an indirect marker of exercise performance, may be useful for monitoring how athletes are responding to changes in training load. The most appropriate method of determining rHRI would seem to be when running at 8 km/h over assessment periods ranging from two to four minutes.

## Data Availability

All reasonable requests for access to the data from this study will be considered by the authors.
